# Effectiveness of COVID-19 vaccines in preventing SARS-CoV-2 infection and hospitalisation, Navarre, Spain, January to April 2021

**DOI:** 10.2807/1560-7917.ES.2021.26.21.2100438

**Published:** 2021-05-27

**Authors:** Iván Martínez-Baz, Ana Miqueleiz, Itziar Casado, Ana Navascués, Camino Trobajo-Sanmartín, Cristina Burgui, Marcela Guevara, Carmen Ezpeleta, Jesús Castilla, Working Group for the Study of COVID-19 in Navarra, Working Group for the Study of COVID-19 in Navarra, Working Group for the Study of COVID-19 in Navarra, Carlos Ibero Esparza, Mercedes Herranz, Irati Arregui, Carmen Martín, Ana Miqueleiz, Ana Navascués, Isabel Polo, Camino Trobajo-Sanmartín, Carmen Ezpeleta, Ingrid Estévez, Igberto Tordoya, Delia Quílez, Francisco Lameiro, Ana Isabel Álvaro, Paula López Moreno, Esther Albéniz, Fernando Elía, Javier Gorricho, Eva Ardanaz, Nieves Ascunce, Maite Arriazu, Fernando Baigorria, Aurelio Barricarte, Cristina Burgui, Itziar Casado, Enrique de la Cruz, Jorge Díaz, María Ederra, Nerea Egüés, Manuel García Cenoz, Marcela Guevara, Nerea Iriarte, Iván Martínez-Baz, Conchi Moreno-Iribas, Carmen Sayón, Juana Vidán, Marian Nuín, Jesús Castilla

**Affiliations:** 1 Instituto de Salud Pública de Navarra, Pamplona, Spain; 2 CIBER Epidemiología y Salud Pública (CIBERESP), Madrid, Spain; 3 Navarra Institute for Health Research (IdiSNA), Pamplona, Spain; 4 Clinical Microbiology Department, Complejo Hospitalario de Navarra, Pamplona, Spain; 5 Members of the working group are listed under the Investigators tab

**Keywords:** COVID-19, SARS-CoV-2, COVID-19 vaccine, cohort study, vaccine effectiveness, close contact

## Abstract

COVID-19 vaccine effectiveness was evaluated in close contacts of cases diagnosed during January–April 2021. Among 20,961 contacts, 7,240 SARS-CoV-2 infections were confirmed, with 5,467 being symptomatic and 559 leading to hospitalisations. Non-brand-specific one and two dose vaccine effectiveness were respectively, 35% (95% confidence interval (CI): 25 to 44) and 66% (95% CI: 57 to 74) against infections, 42% (95% CI: 31 to 52) and 82% (95% CI: 74 to 88) against symptomatic infection, and 72% (95% CI: 47 to 85) and 95% (95% CI: 62 to 99) against COVID-19 hospitalisation. The second dose significantly increased effectiveness. Findings support continuing complete vaccination.

In this study in Navarre, Spain, we estimate the effectiveness of coronavirus disease (COVID-19) vaccines in preventing confirmed severe acute respiratory syndrome coronavirus 2 (SARS-CoV-2) infections, symptomatic confirmed SARS-CoV-2 infections and COVID-19 hospitalisations in adults (≥ 18 years old) who had had close contact to a person with confirmed SARS-CoV-2 infection.

## Study design, setting and information sources

This prospective cohort study included all individuals aged ≥ 18 years covered by the Navarre Health Service, who had been close contacts of laboratory-confirmed COVID-19 cases from January to April 2021.

As part of measures to control COVID-19, all laboratory-confirmed COVID-19 cases, according to the European Union definition [1], were interviewed to identify their close contacts [2]. A close contact of a COVID-19 case was defined as any person who had had high-risk exposure to a confirmed COVID-19 case within a timeframe ranging from 2 days before the onset of symptoms in the case to 10 days after the onset of symptoms, or in the 2 days before the sample, which led to confirmation was taken, to 10 days after the sample was taken for asymptomatic cases [3]. Close contacts were tested by reverse-transcription (RT)-PCR for SARS-CoV-2 in nasopharynx samples initially and 10 days after the last contact. In symptomatic contacts, a positive antigen test within 5 days from the symptom onset was also considered confirmatory of SARS-CoV-2 infection, because these tests have demonstrated very high specificity [4,5]. Close contacts with a positive test for SARS-CoV-2 before January 2021, nursing home residents and those who did not complete the testing protocol were excluded from the study. COVID-19 hospitalisation entailed admission due to laboratory-confirmed COVID-19 for ≥ 24 hours. Age, sex, chronic conditions, healthcare work and contact setting (household or other) were obtained from the enhanced epidemiological surveillance of COVID-19 [2].

During the course of the COVID-19 pandemic, the European Medicines Agency initially authorised three vaccines to control the spread of SARS-CoV-2, i.e. Comirnaty (BNT162b2 mRNA, BioNTech-Pfizer, Mainz, Germany/New York, United States (US)), Moderna COVID-19 Vaccine (mRNA-1273, Moderna, Cambridge, US) and Vaxzevria (ChAdOx1 nCoV-19, Oxford-AstraZeneca, Cambridge, United Kingdom) [6-8]. In Spain, the COVID-19 vaccination campaign started on 27 December 2020 with Comirnaty, followed by the progressive incorporation of Moderna and Vaxzevria vaccines. Vaccination was successively targeted to residents and workers in long-term care facilities, healthcare workers, people with disabilities, individuals aged ≥ 80 years, essential workers, persons with high-risk conditions, and individuals in the general population aged ≥ 60 years [9]. COVID-19 vaccine doses, brand and date of administration were obtained from the regional vaccination register. Each dose was considered potentially effective 14 days after administration [10].

## Statistical analysis

The incidences of SARS-CoV-2 infection, symptomatic COVID-19 and hospitalisation were compared by COVID-19 vaccination status. The same risk period was assigned to everyone in the cohort; therefore, the Cox regression provided estimates of the crude and adjusted relative risks (RR) with 95% confidence intervals (CI) [11]. Adjusted models included age groups, sex, major chronic condition, contact setting (household or other) and month. Contacts who had received the first dose < 14 days before testing were excluded from the analyses. Vaccine effectiveness (VE) was estimated as a percentage: (1 − adjusted RR) × 100. Interaction between vaccination status and age group was tested. The relative effectiveness of two doses vs one dose was also evaluated. Sensitivity analyses in household contacts or considering only RT-PCR results were performed.

## Ethical statement

This study was approved by the Ethical Committee for Clinical Research in Navarre (PI2020/45), which waived the requirement of obtaining informed consent.

## Estimates of the effectiveness of COVID-19 vaccines

The cohort included 20,961 close contacts, 7,240 (34.5%) were confirmed as infected with SARS-CoV-2, with 5,467 (26.1%) having symptomatic infections, and 559 (2.7%) needing hospitalisation for COVID-19. Among the close contacts, 50.4% were household contacts, 78.3% were < 60 years old, 6.5% (n = 1,381) had received any dose of a vaccine and 2.4% (n = 512) had received the second dose ≥ 14 days before testing (Figure and Table 1).

**Figure:**
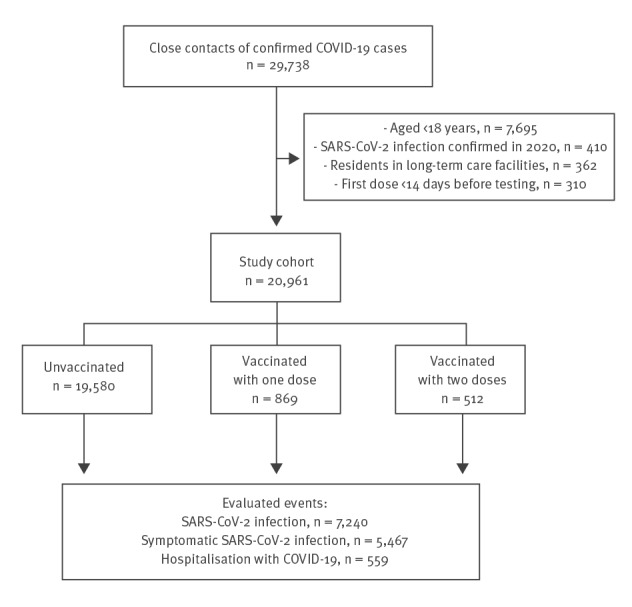
Flowchart of the selection of close contacts of COVID-19 cases for the prospective cohort study on vaccine effectiveness, Navarre, Spain, January–April 2021 (n = 20,961 contacts selected)

COVID-19: coronavirus disease; SARS-CoV-2: severe acute respiratory syndrome coronavirus 2.

**Table 1:** Characteristics of the individuals included in the analysis according to their vaccination status, Navarre, Spain, January–April 2021 (n = 20,961)

Characteristics	Total		Vaccinated		Comirnaty vaccine				Vaxzevria vaccine		Moderna vaccine	
			Any dose		1 dose		2 doses		1 dose		1 dose	2 doses
	Number	%	Number	%	Number	%	Number	%	Number	%	Number	Number
**Age groups (years)**												
18–39	8,008	38.2	377	27.3	27	8.7	124	25.3	217	41.4	4	5
40–59	8,414	40.1	487	35.3	57	18.4	212	43.2	203	38.7	4	11
≥ 60	4,539	21.7	517	37.4	226	72.9	155	31.6	104	19.8	27	5
**Sex**												
Male	10,302	49.1	367	26.6	93	30.0	82	16.7	176	33.6	13	3
Female	10,659	50.9	1014	73.4	217	70.0	409	83.3	348	66.4	22	18
**Major chronic conditions**												
No	14,883	71.0	889	64.4	141	45.5	313	63.7	406	77.5	14	15
Yes	6,078	29.0	492	35.6	169	54.5	178	36.3	118	22.5	21	6
**Contact setting**												
Household	10,574	50.4	649	47.0	141	45.5	246	50.1	231	44.1	18	13
Other	10,387	49.6	732	53.0	169	54.5	245	49.9	293	55.9	17	8
**Month**												
January	6,842	32.6	28	2.0	27	8.7	0	0.0	0	0.0	1	0
February	2,956	14.1	82	5.9	48	15.5	24	4.9	6	1.1	4	0
March	4,331	20.7	347	25.1	82	26.5	120	24.4	128	24.4	10	7
April	6,832	32.6	924	66.9	153	49.4	347	70.7	390	74.4	20	14
**SARS-CoV-2 infection outcomes**												
All infections	7,240	34.5	260	18.8	90	29.0	61	12.4	99	18.9	9	1
Symptomatic COVID-19	5,467	26.1	161	11.7	62	20.0	25	5.1	67	12.8	6	1
COVID-19 hospitalisation	559	2.7	11	0.8	7	2.3	1	0.2	1	0.2	2	0
**Total**	**20,961**	**100**	**1,381**	**100**	**310**	**100**	**491**	**100**	**524**	**100**	**35**	**21**

COVID-19: coronavirus disease; SARS-CoV-2: severe acute respiratory syndrome coronavirus 2.

Comparison of brands should be avoided from this study since they were not randomly distributed.

The incidence of SARS-CoV-2 infection was higher in unvaccinated contacts (35.6%) than in those vaccinated with one or two doses (22.8% and 12.1%, respectively). Incidence of symptomatic COVID-19 was 27.1% in unvaccinated contacts and declined in those vaccinated with one (15.5%) and two doses (5.1%). The whole genome sequencing of 1,256 of the total 7,240 samples of infected contacts (17.3%) showed dominance of the B.1.1.7 lineage (n = 865; 68.9%) (Supplementary Figure).

Of the 1,381 close contacts vaccinated, 801 (58.0%) had received Comirnaty, 524 (37.9%) Vaxzevria and 56 (4.1%) Moderna vaccine. No contact had received the second dose of Vaxzevria because of the longer time interval between doses for this vaccine. Median time from vaccination to diagnosis in those with one and two doses of Comirnaty and one dose of Vaxzevria were 22, 43 and 36 days, respectively. The number of individuals with Moderna vaccine was too small to estimate the VE (Table 1).

COVID-19 VE of one and two doses were, respectively, 35% (95% CI: 25 to 44) and 66% (95% CI: 57 to 74) against SARS-CoV-2 infections. VE estimates improved against symptomatic COVID-19 (42%; 95% CI: 31 to 52, and 82%; 95% CI: 74 to 88, respectively), and were even higher against hospitalised cases (72%; 95% CI: 47 to 85, and 95%; 95% CI: 62 to 99). Among people vaccinated with one dose, the second dose increased significantly the VE in preventing SARS-CoV-2 infection (relative effect 48%; 95% CI: 31 to 61) and symptomatic COVID-19 (relative effect 69%; 95% CI: 53 to 80) (Table 2).

**Table 2:** Effectiveness of COVID-19 vaccination in preventing confirmed SARS-CoV-2 infection and infection outcomes, Navarre, Spain, January–April 2021 (n = 20,961)

Evaluated outcome and vaccination status	Infections/contacts	Crude RR (95% CI)	Adjusted RR (95% CI)^a^	Adjusted VE % (95% CI)^a^
**Confirmed SARS-CoV-2 infection** ^b^				
Unvaccinated	6,980/19,580	Reference	Reference	Reference
Vaccinated with 1 dose	198/869	0.64 (0.56 to 0.74)	0.65 (0.56 to 0.75)	35 (25 to 44)
Vaccinated with 2 doses	62/512	0.34 (0.27 to 0.44)	0.34 (0.26 to 0.43)	66 (57 to 74)
Relative effect of 2 vs 1 dose	62/512 vs 198/869	0.53 (0.40 to 0.71)	0.52 (0.39 to 0.69)	48 (31 to 61)
**Symptomatic COVID-19**				
Unvaccinated	5,306/19,580	Reference	Reference	Reference
Vaccinated with 1 dose	135/869	0.57 (0.48 to 0.68)	0.58 (0.48 to 0.69)	42 (31 to 52)
Vaccinated with 2 doses	26/512	0.19 (0.13 to 0.28)	0.18 (0.12 to 0.26)	82 (74 to 88)
Relative effect of 2 vs 1 dose	26/512 vs 135/869	0.33 (0.22 to 0.50)	0.31 (0.20 to 0.47)	69 (53 to 80)
**Hospitalisation due to COVID-19**				
Unvaccinated	548/19,580	Reference	Reference	Reference
Vaccinated with 1 dose	10/869	0.41 (0.22 to 0.77)	0.28 (0.15 to 0.53)	72 (47 to 85)
Vaccinated with 2 doses	1/512	0.07 (0.01 to 0.50)	0.05 (0.01 to 0.38)	95 (62 to 99)
Relative effect of 2 vs 1 dose	1/512 vs 10/869	0.17 (0.02 to 1.33)	0.19 (0.02 to 1.49)	81 (−49 to 98)
**Symptomatic COVID-19 in 18 to 59 years age group**				
Unvaccinated	4,120/15,558	Reference	Reference	Reference
Vaccinated with 1 dose	65/512	0.48 (0.38 to 0.61)	0.49 (0.38 to 0.63)^c^	51 (37 to 62)
Vaccinated with 2 doses	16/352	0.17 (0.11 to 0.28)	0.16 (0.10 to 0.26)^c^	84 (74 to 90)
Relative effect of 2 vs 1 dose	16/352 vs 65/512	0.36 (0.21 to 0.62)	0.32 (0.18 to 0.55)	68 (45 to 82)
**Symptomatic COVID-19 in ≥ 60 years age group**				
Unvaccinated	1,186/4,022	Reference	Reference	Reference
Vaccinated with 1 dose	70/357	0.67 (0.52 to 0.85)	0.70 (0.55 to 0.90)^c^	30 (10 to 45)
Vaccinated with 2 doses	10/160	0.21 (0.11 to 0.40)	0.23 (0.12 to 0.44)^c^	77 (56 to 88)
Relative effect of 2 vs 1 dose	10/160 vs 70/357	0.32 (0.16 to 0.62)	0.33 (0.17 to 0.65)	67 (35 to 83)

CI: confidence interval; COVID-19: coronavirus disease; RR:  relative risk; SARS-CoV-2: severe acute respiratory syndrome coronavirus 2; VE: vaccine effectiveness.

^a^ RR adjusted by age groups (18–39, 40–59 and ≥ 60 years), sex, contact setting (household or other), major chronic conditions and month.

^b^ Asymptomatic and symptomatic COVID-19.

^c^ Interaction between age group and vaccination with one dose (p = 0.065) and vaccination with two doses (p = 0.375).

The VE against symptomatic COVID-19 was higher in people aged 18–59 years than in those aged ≥ 60 years for one dose (51%; 95% CI: 37 to 62, vs 30%; 95% CI: 10 to 45, p_interaction_ = 0.065) and two doses (84%; 95% CI: 74 to 90, vs 77%; 95% CI: 56 to 88, p_interaction_ = 0.375), although these differences were not statistically significant (Table 2). The relative effect of two doses vs one was similar in both age groups. Sensitivity analyses limited to household contacts or considering only RT-PCR results showed similar estimates (Supplementary Tables S1 and S2).

In people aged 18–59 years, the effectiveness of Comirnaty against symptomatic COVID-19 was 50% (95% CI: 12 to 72) for one dose and 85% (95% CI: 74 to 91) for two doses, while in people aged ≥ 60 years the estimates were 20% (95% CI:−7 to 40) and 76% (95% CI: 55 to 87), respectively (Table 3). In people vaccinated with one dose of Comirnaty, the second dose increased the VE in preventing SARS-CoV-2 infection (relative effect 56%; 95% CI: 39 to 68), and symptomatic COVID-19 (relative effect 74%; 95% CI: 59 to 84).

**Table 3:** Effectiveness of Vaxzevria^a^ vaccine and Comirnaty^b^ vaccine in preventing confirmed SARS-CoV-2 infection and infection outcomes, Navarre, Spain, January–April 2021 (n = 20,905)^c^

Evaluated outcome and vaccination status	Infections/contacts	Crude RR (95% CI)	Adjusted RR (95% CI)^d^	Adjusted VE % (95% CI)^d^
**Confirmed SARS-CoV-2 infection** ^e^				
Unvaccinated	6,980/19,580	Reference	Reference	Reference
Vaxzevria with 1 dose	99/524	0.53 (0.44 to 0.65)	0.56 (0.46 to 0.69)	44 (31 to 54)
Comirnaty with 1 dose	90/310	0.81 (0.66 to 1.00)	0.79 (0.64 to 0.97)	21 (3 to 36)
Comirnaty with 2 doses	61/491	0.35 (0.27 to 0.45)	0.35 (0.27 to 0.44)	65 (56 to 73)
Relative effect of 2 vs 1 dose of Comirnaty	61/491 vs 90/310	0.43 (0.31 to 0.59)	0.44 (0.32 to 0.61)	56 (39 to 68)
**Symptomatic COVID-19**				
Unvaccinated	5,306/19,580	Reference	Reference	Reference
Vaxzevria with 1 dose	67/524	0.47 (0.37 to 0.60)	0.50 (0.39 to 0.63)	50 (37 to 61)
Comirnaty with 1 dose	62/310	0.74 (0.58 to 0.95)	0.70 (0.55 to 0.90)	30 (10 to 45)
Comirnaty with 2 doses	25/491	0.19 (0.13 to 0.28)	0.18 (0.12 to 0.27)	82 (73 to 88)
Relative effect of 2 vs 1 dose of Comirnaty	25/491 vs 62/310	0.26 (0.16 to 0.41)	0.26 (0.16 to 0.41)	74 (59 to 84)
**Hospitalisation due to COVID-19 **				
Unvaccinated	548/19,580	Reference	Reference	Reference
Vaxzevria with 1 dose	1/524	0.07 (0.01 to 0.49)	0.08 (0.01 to 0.54)	92 (46 to 99)
Comirnaty with 1 dose	7/310	0.80 (0.38 to 1.70)	0.35 (0.17 to 0.75)	65 (25 to 83)
Comirnaty with 2 doses	1/491	0.07 (0.01 to 0.52)	0.06 (0.01 to 0.40)	94 (60 to 99)
Relative effect of 2 vs 1 dose of Comirnaty	1/491 vs 7/310	0.09 (0.01 to 0.73)	0.16 (0.02 to 1.29)	84 (–29 to 98)
**Symptomatic COVID-19 in 18 to 59 year age group**				
Unvaccinated	4,120/15,558	Reference	Reference	Reference
Vaxzevria with 1 dose	53/420	0.48 (0.36 to 0.63)	0.50 (0.38 to 0.66)^f^	50 (34 to 62)
Comirnaty with 1 dose	12/84	0.54 (0.31 to 0.95)	0.50 (0.28 to 0.88)^f^	50 (12 to 72)
Comirnaty with 2 doses	15/336	0.17 (0.10 to 0.28)	0.15 (0.09 to 0.26)^f^	85 (74 to 91)
Relative effect of 2 vs 1 dose of Comirnaty	15/336 vs 12/84	0.31 (0.15 to 0.67)	0.31 (0.15 to 0.67)	69 (33 to 85)
**Symptomatic COVID-19 in ≥ 60 year age group**				
Unvaccinated	1,186/4,022	Reference	Reference	Reference
Vaxzevria with 1 dose	14/104	0.46 (0.27 to 0.77)	0.47 (0.28 to 0.81)^f^	53 (19 to 72)
Comirnaty with 1 dose	50/226	0.75 (0.57 to 1.00)	0.80 (0.60 to 1.07)^f^	20 (−7 to 40)
Comirnaty with 2 doses	10/155	0.22 (0.12 to 0.41)	0.24 (0.13 to 0.45)^f^	76 (55 to 87)
Relative effect of 2 vs 1 dose of Comirnaty	10/155 vs 50/226	0.29 (0.15 to 0.58)	0.30 (0.15 to 0.60)	70 (40 to 85)

CI: confidence interval; COVID-19: coronavirus disease; RR:  relative risk; SARS-CoV-2: severe acute respiratory syndrome coronavirus 2; VE: vaccine effectiveness.

^a^ Vaxzevria (ChAdOx1 nCoV-19, Oxford-AstraZeneca, Cambridge, United Kingdom).

^b^ Comirnaty (BNT162b2 mRNA, BioNTech-Pfizer, Mainz, Germany/New York, United States).

^c^ The number of individuals who received the Moderna COVID-19 Vaccine (mRNA-1273, Moderna, Cambridge, United States) was too low to estimate vaccine effectiveness.

^d^ RR adjusted by age groups (18–39, 40–59 and ≥ 60 years), sex, contact setting (household or other), major chronic conditions and month.

^e^ Asymptomatic and symptomatic COVID-19 infections.

^f^ Interaction between age group and vaccination with one dose of Vaxzevria (p = 0.809), vaccination with one dose of Comirnaty (p = 0.179) and vaccination with two doses of Comirnaty (p = 0.327).

Comparison of brands should be avoided from this study since they were not randomly distributed.

The effectiveness of one dose of Vaxzevria was similar in people aged 18–59 and ≥ 60 years: 50% (95% CI: 34 to 62) and 53% (95% CI: 19 to 72, p_interaction_ = 0.809), respectively (Table 3).

## Discussion

By 2 May 2021, the COVID-19 pandemic has caused over 3.5 million cases and 78,000 deaths in Spain [12]. The European Medicines Agency initially authorised three vaccines to control the COVID-19 pandemic in the European Union, i.e. Comirnaty, Moderna and Vaxzevria that had demonstrated high efficacy [6-8]. Since efficacy does not always guarantee the VE in real-life conditions, observational studies covering diverse populations, vaccine brands, study designs and a longer follow-up are necessary [10]. VE studies have the challenge of bias control; because people vaccinated might be more careful in avoiding risk exposures. As close contacts of COVID-19 cases have had a known risk exposure, the comparison of COVID-19 incidence between vaccinated and unvaccinated close contacts is an ideal design to assess the COVID-19 VE.

Our results suggest a moderate effectiveness of Comirnaty and Vaxzevria vaccines in preventing SARS-CoV-2 infection, and estimates increased against symptomatic and hospitalised COVID-19. VE was moderate in people with one dose and was higher after the second dose, especially against hospitalisations. The second dose significantly improved the preventive effect of the first dose. However, we found estimates slightly lower than those reported in clinical trials [6-8] and in some recent observational studies [13-19]. These differences could be explained because some studies may not represent the general population (e.g. studies in healthcare workers), include an indirect effect in highly vaccinated groups, consider a short follow-up, or might be affected by residual bias due to lower risk exposure of vaccinated subjects.

Our results highlight several aspects with relevant practical implications. The VE of Comirnaty seemed to be lower in older people than in young adults. As the risk of vaccine failures increases with age, additional preventive measures should be maintained and community immunity should be guaranteed around vulnerable persons. The effectiveness of one dose of Vaxzevria was moderate in preventing infection in adults aged < 70 years in < 4 months after administration. In our study, complete vaccination with Comirnaty demonstrated very high effectiveness in preventing COVID-19 hospitalisation, which may have a very positive impact on the healthcare system. However, as the VE in preventing confirmed infection was moderate, the study and quarantine of complete vaccinated individuals when they are close contacts of confirmed cases may remain recommendable, since the possibility of acquiring and transmitting the infection has not been totally excluded.

Compared with the test-negative and specific-cohort designs, the cohort of close contacts has advantages for bias control, since participants had a similar risk exposure regardless their vaccination status, and provided a good representativeness of the general population [20]. Only close contacts tested for COVID-19 were included in this study.

This study has some limitations. The proportion of vaccinated persons was low and results are preliminary. As no contact had received two doses of Vaxzevria, this effectiveness could not be estimated. The study included individuals with different chance for vaccination, but age, comorbidity and month were included in the analysis. Symptomatic patients with positive antigen test were included; however, the sensitivity analysis including only RT-PCR results provided similar estimates. Some close contacts who had received the first dose < 14 days before testing could have been unnecessarily excluded. Comparison of brands should be avoided from this study since they were not randomly distributed. VE estimates were obtained under specific epidemiological and vaccination conditions and may vary in other populations and moments.

## Conclusions

COVID-19 VE was moderate in preventing SARS-CoV-2 infection and was higher against symptomatic and hospitalised cases. One dose of COVID-19 vaccine showed a moderate effect and two doses reached a high effectiveness in reducing symptomatic COVID-19 and hospitalisation. The second dose significantly improved the effectiveness. These findings highlight the benefit of immunization against SARS-CoV-2 and support continuing to complete vaccination.

## Working Group for the Study of COVID-19 in Navarra

Carlos Ibero Esparza, Mercedes Herranz, Irati Arregui, Carmen Martín, Ana Miqueleiz, Ana Navascués, Isabel Polo, Camino Trobajo-Sanmartín, Carmen Ezpeleta (Complejo Hospitalario de Navarra, Pamplona, España); Ingrid Estévez, Igberto Tordoya, Delia Quílez (Hospital Reina Sofía de Tudela); Francisco Lameiro, Ana Isabel Álvaro (Hospital García Orcoyen de Estella); Paula López Moreno, Esther Albéniz, Fernando Elía, Javier Gorricho (Servicio Navarro de Salud-Osasunbidea, Pamplona, Spain); Eva Ardanaz, Nieves Ascunce, Maite Arriazu, Fernando Baigorria, Aurelio Barricarte, Cristina Burgui, Itziar Casado, Enrique de la Cruz, Jorge Díaz, María Ederra, Nerea Egüés, Manuel García Cenoz, Marcela Guevara, Nerea Iriarte, Iván Martínez-Baz, Conchi Moreno-Iribas, Carmen Sayón, Juana Vidán, Marian Nuín and Jesús Castilla (Instituto de Salud Pública y Laboral de Navarra – IdiSNA – CIBERESP, Pamplona, Spain).

## Supplementary Data

279000 Supplementary_Material
