# Supplementary material for: Effectiveness of COVID-19 vaccines in preventing SARS-CoV-2 infection and hospitalisation, Navarre, Spain, January to April 2021
Source: Euro Surveill. 2021 May 27;26(21):2100438. doi: 10.2807/1560-7917.ES.2021.26.21.2100438 (PMC8161727; doi:10.2807/1560-7917.ES.2021.26.21.2100438)

SUPPLEMENTARY MATERIAL

This supplementary material is hosted by *Eurosurveillance* as supporting information alongside the article “Effectiveness of COVID-19 vaccines in preventing SARS-CoV-2 infection and hospitalisation, Navarre, Spain, January to April 2021” on behalf of the authors who remain responsible for the accuracy and appropriateness of the content. The same standards for ethics, copyright, attributions and permissions as for the article apply. Supplements are not edited by *Eurosurveillance* and the journal is not responsible for the maintenance of any links or email addresses provided therein.

Supplementary Table S1. Effectiveness of COVID-19 vaccination in preventing confirmed SARS-CoV-2 outcomes among household close contacts. Navarre, Spain, January–April 2021 (n=10,574)

Evaluated outcome and vaccination status	Infections/ contacts	Crude relative risk (95% CI)	Adjusted relative risk (95% CI)[a]	Adjusted VE, % (95% CI)[a]
**Confirmed SARS-CoV-2 infection** [b]				
Unvaccinated	4,987/9,925	Reference	Reference	Reference
Vaccinated with 1 dose	136/390	0.69 (0.59-0.82)	0.65 (0.55-0.78)	35 (22 to 45)
Vaccinated with 2 doses	44/259	0.34 (0.25-0.46)	0.33 (0.24-0.44)	67 (56 to 76)
Relative effect of 2 vs 1 dose		0.49 (0.35-0.68)	0.50 (0.36-0.70)	50 (30 to 64)
**Symptomatic COVID-19**				
Unvaccinated	3,883/9,925	Reference	Reference	Reference
Vaccinated with 1 dose	96/390	0.63 (0.51-0.77)	0.59 (0.48-0.72)	41 (28 to 52)
Vaccinated with 2 doses	20/259	0.20 (0.13-0.31)	0.19 (0.12-0.29)	81 (71 to 88)
Relative effect of 2 vs 1 dose		0.31 (0.19-0.51)	0.32 (0.20-0.51)	68 (49 to 80)
**Hospitalisation due to COVID-19**				
Unvaccinated	390/9,925	Reference	Reference	Reference
Vaccinated with 1 dose	7/390	0.46 (0.22-0.96)	0.28 (0.13-0.59)	72 (41 to 87)
Vaccinated with 2 doses	1/259	0.10 (0.01-0.70)	0.07 (0.01-0.53)	93 (47 to 99)
Relative effect of 2 vs 1 dose		0.22 (0.03-1.75)	0.27 (0.03-2.16)	73 (-116 to 97)
**Symptomatic COVID-19 in 18-59 years age group**				
Unvaccinated	3,031/8,032	Reference	Reference	Reference
Vaccinated with 1 dose	50/233	0.57 (0.43-0.75)	0.55 (0.42-0.73)	45 (27 to 58)
Vaccinated with 2 doses	12/196	0.16 (0.09-0.29)	0.15 (0.09-0.27)	85 (73 to 91)
Relative effect of 2 vs 1 dose		0.29 (0.15-0.54)	0.28 (0.15-0.52)	72 (48 to 85)
**Symptomatic COVID-19 in ≥60 years age group**				
Unvaccinated	852/1,893	Reference	Reference	Reference
Vaccinated with 1 dose	46/157	0.65 (0.48-0.88)	0.62 (0.46-0.85)	38 (15 to 54)
Vaccinated with 2 doses	8/63	0.28 (0.14-0.57)	0.27 (0.13-0.54)	73 (46 to 87)
Relative effect of 2 vs 1 dose		0.43 (0.21-0.92)	0.43 (0.20-0.91)	57 (8 to 79)

CI: confidence interval; COVID-19: coronavirus disease; SARS-CoV-2: severe acute respiratory syndrome coronavirus 2; VE: vaccine effectiveness.

[a] Relative risk adjusted by age groups (18-39, 40-59, and ≥60 years), sex, major chronic conditions and month.

[b] Asymptomatic and symptomatic SARS-CoV-2 infections.

Supplementary Table S2. Effectiveness of COVID-19 vaccination in preventing confirmed SARS-CoV-2 outcomes assessed exclusively in close contacts tested by RT-PCR. Navarre, Spain, January–April 2021 (n=19,376)

**Evaluated outcome and vaccination status**	**Infections/ contacts**	**Crude relative risk (95% CI)**	**Adjusted relative risk (95% CI)[a]**	**Adjusted VE, % (95% CI)[a]**
**Confirmed SARS-CoV-2 infection [b]**				
Unvaccinated	5,431/18,031	Reference	Reference	Reference
Vaccinated with 1 dose	166/837	0.66 (0.56-0.77)	0.66 (0.57-0.78)	34 (22 to 43)
Vaccinated with 2 doses	58/508	0.38 (0.29-0.49)	0.37 (0.29-0.48)	63 (52 to 71)
Relative effect of 2 vs 1 dose		0.58 (0.43-0.78)	0.56 (0.41-0.75)	44 (25 to 59)
**Symptomatic COVID-19**				
Unvaccinated	3,768/18,031	Reference	Reference	Reference
Vaccinated with 1 dose	103/837	0.59 (0.48-0.72)	0.58 (0.48-0.71)	42 (29 to 52)
Vaccinated with 2 doses	23/508	0.22 (0.14-0.33)	0.20 (0.13-0.31)	80 (69 to 87)
Relative effect of 2 vs 1 dose		0.37 (0.23-0.58)	0.35 (0.22-0.55)	65 (45 to 78)
**Hospitalisation due to COVID-19**				
Unvaccinated	413/18,031	Reference	Reference	Reference
Vaccinated with 1 dose	9/837	0.47 (0.24-0.91)	0.32 (0.16-0.62)	68 (38 to 84)
Vaccinated with 2 doses	1/508	0.09 (0.01-0.61)	0.07 (0.01-0.47)	93 (53 to 99)
Relative effect of 2 vs 1 dose		0.18 (0.02-1.45)	0.20 (0.03-1.63)	80 (-63 to 97)
**Symptomatic COVID-19 in 18-59 years age group**				
Unvaccinated	2,895/14,323	Reference	Reference	Reference
Vaccinated with 1 dose	50/497	0.50 (0.38-0.66)	0.50 (0.38-0.67)	50 (33 to 62)
Vaccinated with 2 doses	13/348	0.19 (0.11-0.32)	0.17 (0.10-0.29)	83 (71 to 90)
Relative effect of 2 vs 1 dose		0.37 (0.20-0.68)	0.33 (0.18-0.60)	67 (40 to 82)
**Symptomatic COVID-19 in ≥60 years age group**				
Unvaccinated	873/3,708	Reference	Reference	Reference
Vaccinated with 1 dose	53/340	0.66 (0.50-0.87)	0.72 (0.54-0.96)	28 (4 to 46)
Vaccinated with 2 doses	10/160	0.27 (0.14-0.50)	0.30 (0.16-0.57)	70 (43 to 84)
Relative effect of 2 vs 1 dose		0.40 (0.20-0.79)	0.42 (0.21-0.83)	58 (17 to 79)

CI: confidence interval; COVID-19: coronavirus disease; SARS-CoV-2: severe acute respiratory syndrome coronavirus 2; VE: vaccine effectiveness.

[a] Relative risk adjusted by age groups (18-39, 40-59, and ≥60 years), sex, contact setting (household or other), major chronic conditions and month.

[b] Asymptomatic and symptomatic SARS-CoV-2 infections.

Supplementary Figure. Results of the whole genome sequencing of SARS-CoV-2 among patients confirmed infection in the study. Navarre, Spain, January to April 2021

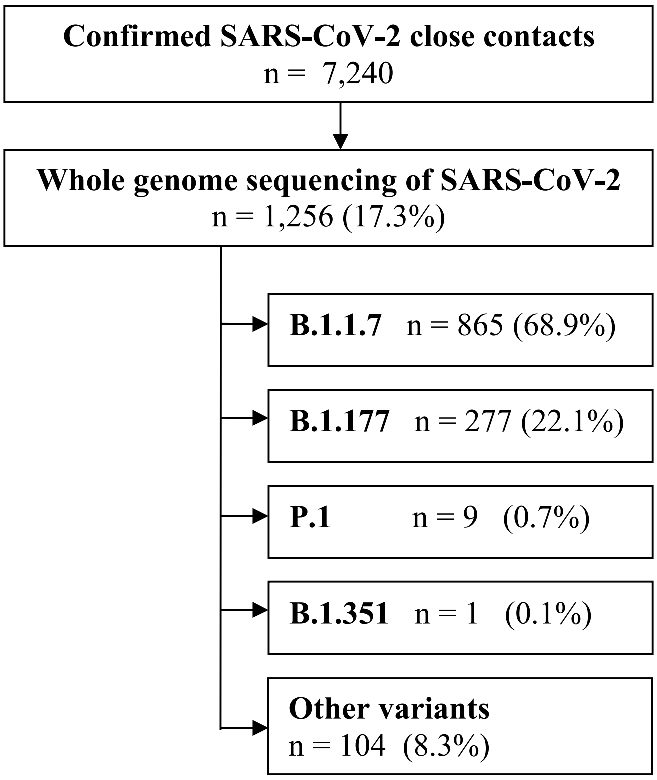

Supplement: Supplementary_Material [file 2100438_Supplementary_Material.pdf]
